# Supplementary material for: The causal role of circulating inflammatory markers in osteoporosis: a bidirectional Mendelian randomized study
Source: Front Immunol. 2024 Jul 18;15:1412298. doi: 10.3389/fimmu.2024.1412298 (PMC11291241; doi:10.3389/fimmu.2024.1412298)
Supplement: Supplementary file 1 [file DataSheet_1.zip › supplementary material/CIM-OSTEOPOROSIS /GCST90274818.csv_leave_one_out.pdf]

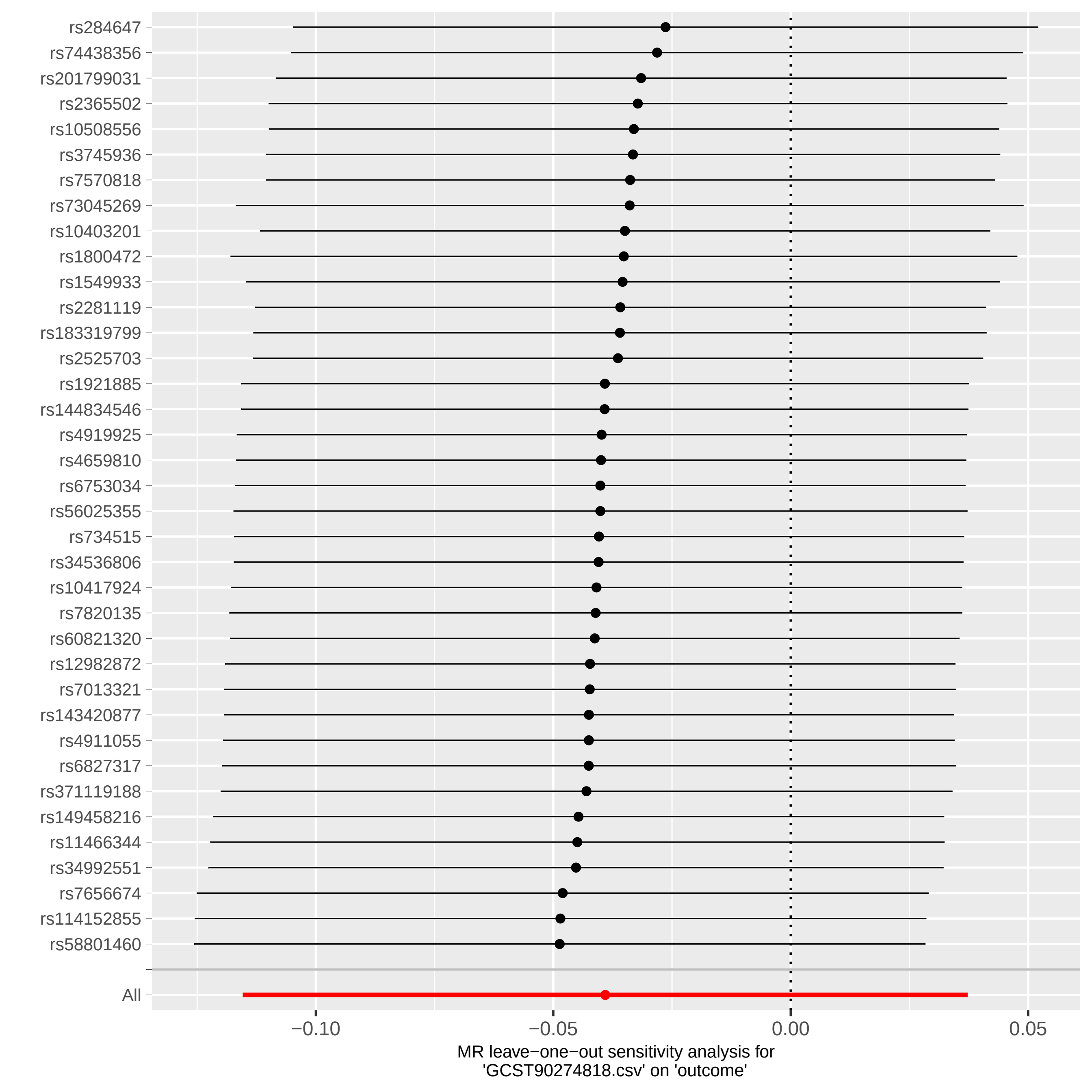

rs284647
rs74438356
rs201799031
rs2365502
rs10508556
rs3745936
rs7570818
rs73045269
rs10403201
rs1800472
rs1549933
rs2281119
rs183319799
rs2525703
rs1921885
rs144834546
rs4919925
rs4659810
rs6753034
rs56025355
rs734515
rs34536806
rs10417924
rs7820135
rs60821320
rs12982872
rs7013321
rs143420877
rs4911055
rs6827317
rs371119188
rs149458216
rs11466344
rs34992551
rs7656674
rs114152855
rs58801460
All
−0.10
−0.05
0.00
0.05
MR leave−one−out sensitivity analysis for
'GCST90274818.csv' on 'outcome'
